# Risk factor analysis for ceftriaxone-associated liver dysfunction in older patients

**DOI:** 10.1186/s40780-026-00554-6

**Published:** 2026-02-11

**Authors:** Tomohiko Tagashira, Ryoya Odawara, Naohito Suga, Rikako Nakamura, Makoto Tagashira, Kohei Minematsu

**Affiliations:** Department of Pharmacy, Innoshima-Ishikai Hospital, 1962 Nakanosho-cho, Innoshima, Onomichi-shi, Hiroshima, Japan

**Keywords:** Ceftriaxone, Liver dysfunction, Alanine aminotransferase, C-reactive protein

## Abstract

**Background:**

Information on ceftriaxone (CTRX)-associated liver dysfunction in older patients remains limited. This study, investigated the risk factors for CTRX-associated liver dysfunction in patients aged ≥65 years.

**Methods:**

We conducted a retrospective chart review of the medical records of 105 hospitalized patients aged ≥65 years who received CTRX at Innoshima-Ishikai Hospital. Variables significantly associated with liver dysfunction in univariate analyses were entered into a multivariate logistic regression model to identify independent risk factors. Cutoff values were determined using receiver operating characteristic curve analysis. The incidence of liver dysfunction was compared according to the number of identified risk factors. Fisher’s exact test was used for comparisons between groups.

**Results:**

In univariate analyses, alanine aminotransferase (ALT) and C-reactive protein (CRP) levels were significantly associated with liver dysfunction (*p* < 0.05). Multivariate logistic regression identified ALT as an independent risk factor (odds ratio [OR] 1.14; 95% confidence interval [CI]: 1.04–1.24, *p* = 0.003). CRP was also significantly associated with liver dysfunction, although the effect size was small (OR 1.07; 95% CI: 1.00–1.14, *p* = 0.043). The optimal cutoff values were 11 U/L for ALT and 5.9 mg/dL for CRP. The incidence of liver dysfunction was 0% in patients with no risk factors, 10.7% in those with elevated ALT only, 8.3% in those with elevated CRP only, and 50% in those with both elevated ALT and CRP. Patients with both risk factors had a significantly higher incidence of liver dysfunction than those in the other groups (*p* < 0.001).

**Conclusion:**

Among patients aged ≥ 65 years, elevated baseline ALT (≥ 11 U/L) and CRP (≥ 5.9 mg/dL) levels were associated with an increased risk of CTRX-associated liver dysfunction. Careful monitoring of liver function during and after CTRX administration is therefore recommended in this population.

## Background

Ceftriaxone (CTRX) is a broad-spectrum antibiotic with activity against gram-positive and gram-negative aerobic bacteria as well as anaerobes. Compared with other cephalosporins, it demonstrates favorable tissue penetration into the cerebrospinal fluid, bile, ascites, and sputum, and has a relatively long plasma half-life of 7–8 h, allowing once-daily administration. Therefore, CTRX is widely used in the treatment of various infectious diseases. Nevertheless, cases of severe hepatitis, including fulminant hepatitis, and liver dysfunction characterized by elevations in aspartate aminotransferase (AST), alanine aminotransferase (ALT), and γ-glutamyl transferase levels, as well as the occurrence of jaundice, have been reported [1].

When drug-induced liver dysfunction occurs, standard management involves discontinuation of the suspected drug and substitution with an alternative agent. However, such changes may compromise the effectiveness of infection treatment. Therefore, identifying risk factors for CTRX-associated liver dysfunction prior to administration would enable clinicians to implement appropriate measures, such as closer monitoring of liver function or reconsideration of antibiotic selection.

Although several studies have reported potential risk factors for CTRX-associated liver dysfunction, including administration of high-doses (4 g/day) [2], concomitant use of hepatically metabolized drugs [3], and associations with the albumin–bilirubin score [4], most of these studies were conducted in adult populations that included younger patients. Therefore, risk factors, specifically in older patients aged ≥ 65 years, have not been sufficiently investigated. Moreover, because CTRX is frequently used to treat infections in older patients, information on risk factors for CTRX-associated liver dysfunction in this population is clinically valuable.

Amemiya et al. identified risk factors for tazobactam/piperacillin-induced liver injury using clinical data obtained before administration, and reported that this approach requires no special testing and provides a favorable cost-benefit balance [5]. Given that CTRX is also a β-lactam antibiotic, we conducted the present study to identify risk factors for CTRX-associated liver dysfunction using pre-administration clinical data.

## Methods

### Study patients and design

We conducted a retrospective chart review of the medical records of hospitalized patients aged ≥ 65 years who received CTRX at Innoshima-Ishikai Hospital between May 2022 and April 2025. The collected variables included age, sex, duration of CTRX administration, daily CTRX dose, cumulative CTRX dose, concomitant medications previously reported to be associated with CTRX-associated liver dysfunction (acetaminophen and azithromycin) [3], and clinical laboratory values obtained immediately before administration. Fifteen laboratory parameters routinely measured in clinical practice were analyzed: blood urea nitrogen, serum creatinine, AST, ALT, lactate dehydrogenase, total bilirubin (T-Bil), albumin, C-reactive protein (CRP), sodium, chloride, potassium, white blood cell count, red blood cell count, hemoglobin, and platelet counts.

### Definition of liver dysfunction

Liver dysfunction was defined as an elevation in ALT levels to Grade 1 or higher according to the Common Terminology Criteria for Adverse Events version 5.0, Japanese Clinical Oncology Group (CTCAE v5.0-JCOG), occurring after CTRX administration.

### Exclusion criteria

- Patients without ALT measurements before and after CTRX administration
- Patients whose ALT level was elevated to Grade 1 or higher according to laboratory tests performed immediately prior to CTRX administration
- Patients who were switched to CTRX from another antimicrobial agent

### Statistical analysis

Comparisons between the liver dysfunction and non–liver dysfunction groups, were performed using the Mann–Whitney *U* test for continuous variables, and Fisher’s exact test for categorical variables. Changes in ALT levels before and after CTRX administration within the same patients were assessed using the Wilcoxon signed-rank test.

Multivariate analysis, was conducted using logistic regression, with variables showing *p* < 0.05 in univariate analyses entered as explanatory variables. Odds ratios (ORs), 95% confidence intervals (CIs), and variance inflation factors (VIFs) were calculated. Risk factors identified by logistic regression were further evaluated using receiver operating characteristic (ROC) curve analysis to determine cutoff values, area under the curve (AUC), sensitivity, and specificity. The incidence of liver dysfunction according to the number of risk factors was compared using Fisher’s exact test. Bonferroni correction was applied to adjust for multiple comparisons; because six tests were performed, the significance threshold was adjusted to 0.05/6 = 0.008. All statistical analyses were conducted using EZR version 1.68 [6].

## Results

### Patient characteristics and univariate analysis

**Fig. 1:**
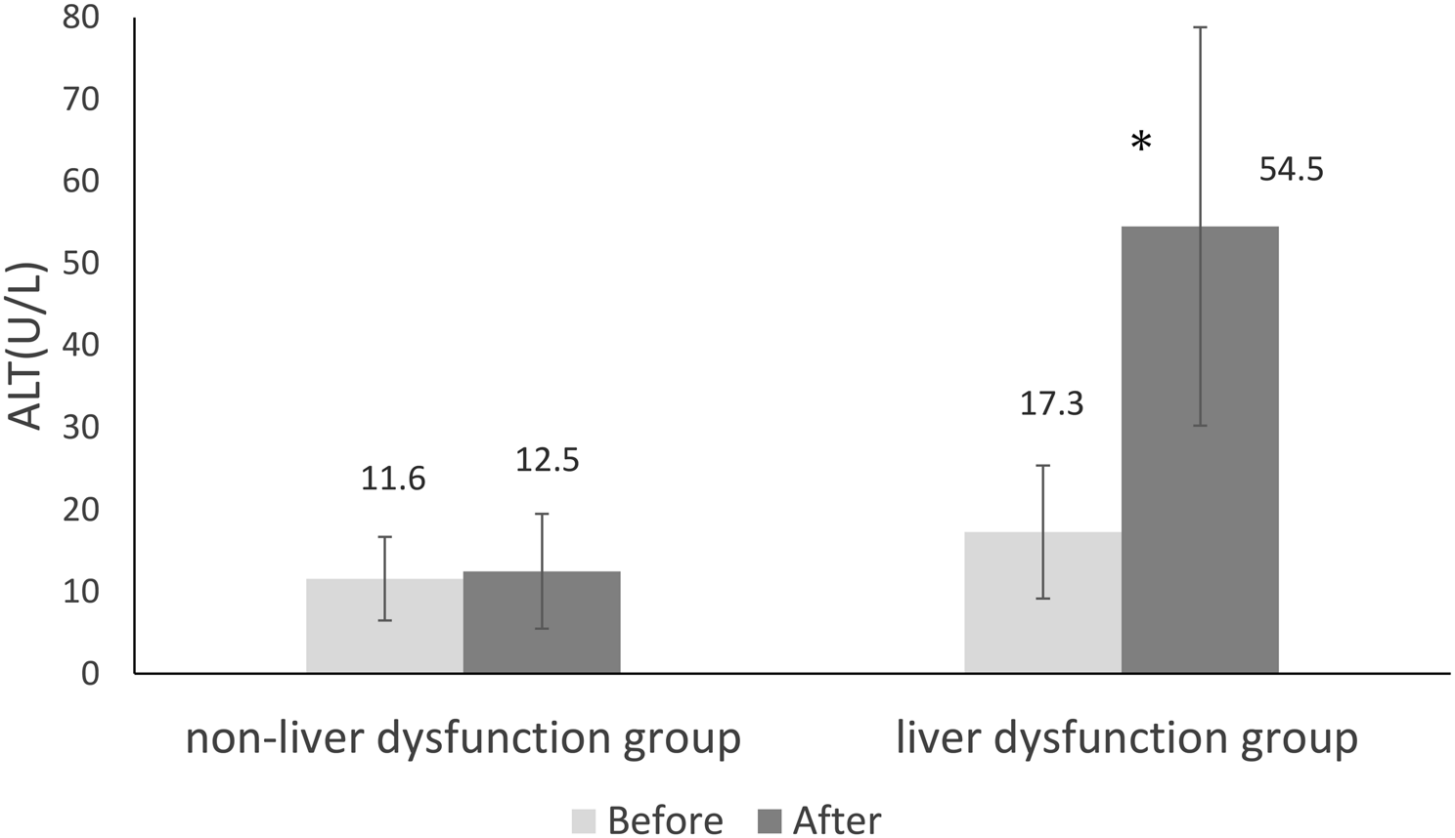
Comparison of ALT levels before and after CTRX administration (mean ± S.D.). *p < 0.001 (Wilcoxon signed-rank test vs. before administration)

A total of 105 hospitalized patients aged ≥ 65 years who received CTRX at Innoshima-Ishikai Hospital between May 2022 and April 2025 were included in the analysis, after applying the exclusion criteria. Liver dysfunction developed in 19 patients, yielding an incidence of 18.1% (19/105). In the liver dysfunction group, ALT levels increased significantly after CTRX administration (Fig. 1). The median time to onset of liver dysfunction was 8 days (interquartile range, 6.5–9.5 days); moreover, 58% of patients developed liver dysfunction within 8 days. Table 1 presents patient characteristics and the results of the univariate analysis comparing the liver dysfunction and non–liver dysfunction groups. No significant between-group differences were observed in age, sex, duration of CTRX administration, cumulative CTRX dose, or concomitant medication use. The daily CTRX dose was 2 g/day in 101 patients (96% of the cohort). Significant differences were observed in ALT (*p* = 0.001), AST (*p* = 0.046), and CRP (*p* = 0.009) levels between groups, whereas no other laboratory parameters differed significantly.

**Table 1:**
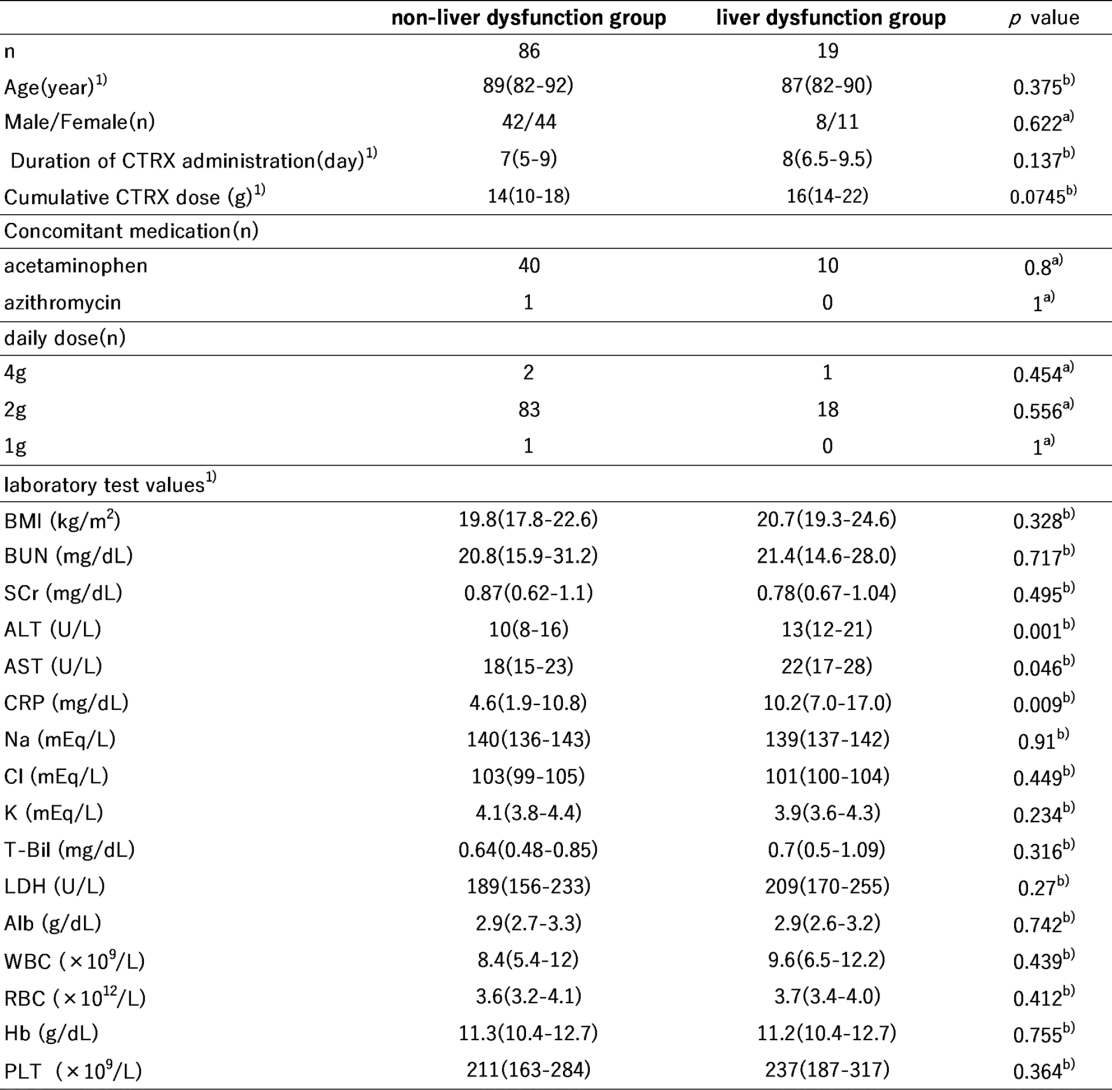
Patient characteristics and univariate analysis

1) Data are presented as medians (interquartile ranges)

a) Fisher’s exact test

b) Mann–Whitney *U* test

### Multivariate analysis and ROC analysis

Multivariate logistic regression analysis was performed using liver dysfunction as the dependent variable and ALT and CRP, which were significant in the univariate analysis (*p* < 0.05), as independent variables. Although AST was also significantly associated with liver dysfunction in the univariate analysis, ALT was selected for inclusion because it is predominantly present in hepatocytes and considered a liver-specific marker, whereas AST is non-specific and present in multiple organs, including the liver, heart, and skeletal muscle [7]. Additionally, to maintain an events-per-variable ratio of ≥ 10, the number of independent variables was limited to two, therefore, ALT, which demonstrated a stronger association (*p* = 0.001), was included in the model. ALT was identified as an independent risk factor (OR 1.14, 95% CI: 1.04–1.24, *p* = 0.003). CRP showed borderline statistical significance (OR 1.07, 95% CI: 1.00–1.14, *p* = 0.043). VIF for each independent variable was 1.00, indicating no multicollinearity (Table 2). Furthermore, ROC curve analysis was performed to determine optimal cutoff values for ALT and CRP in predicting liver dysfunction. The cutoff value for ALT was 11 U/L (AUC 0.744, 95% CI. 0.63–0.86, specificity 0.55, sensitivity 0.90), and that for CRP was 5.9 mg/dL (AUC 0.693, 95% CI. 0.56–0.83, specificity 0.58, sensitivity 0.84) (Table 3).

**Table 2:**
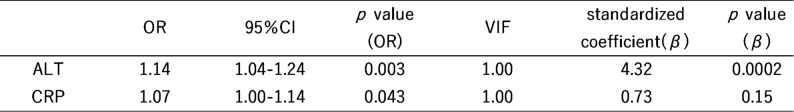
Logistic regression analysis of factors associated with liver dysfunction

**Table 3:**
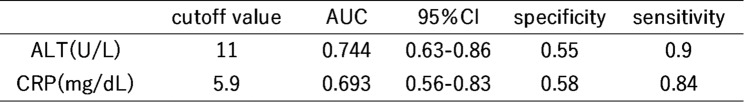
ROC analysis for ALT and CRP

### Incidence of liver dysfunction according to the number of risk factors

**Fig. 2:**
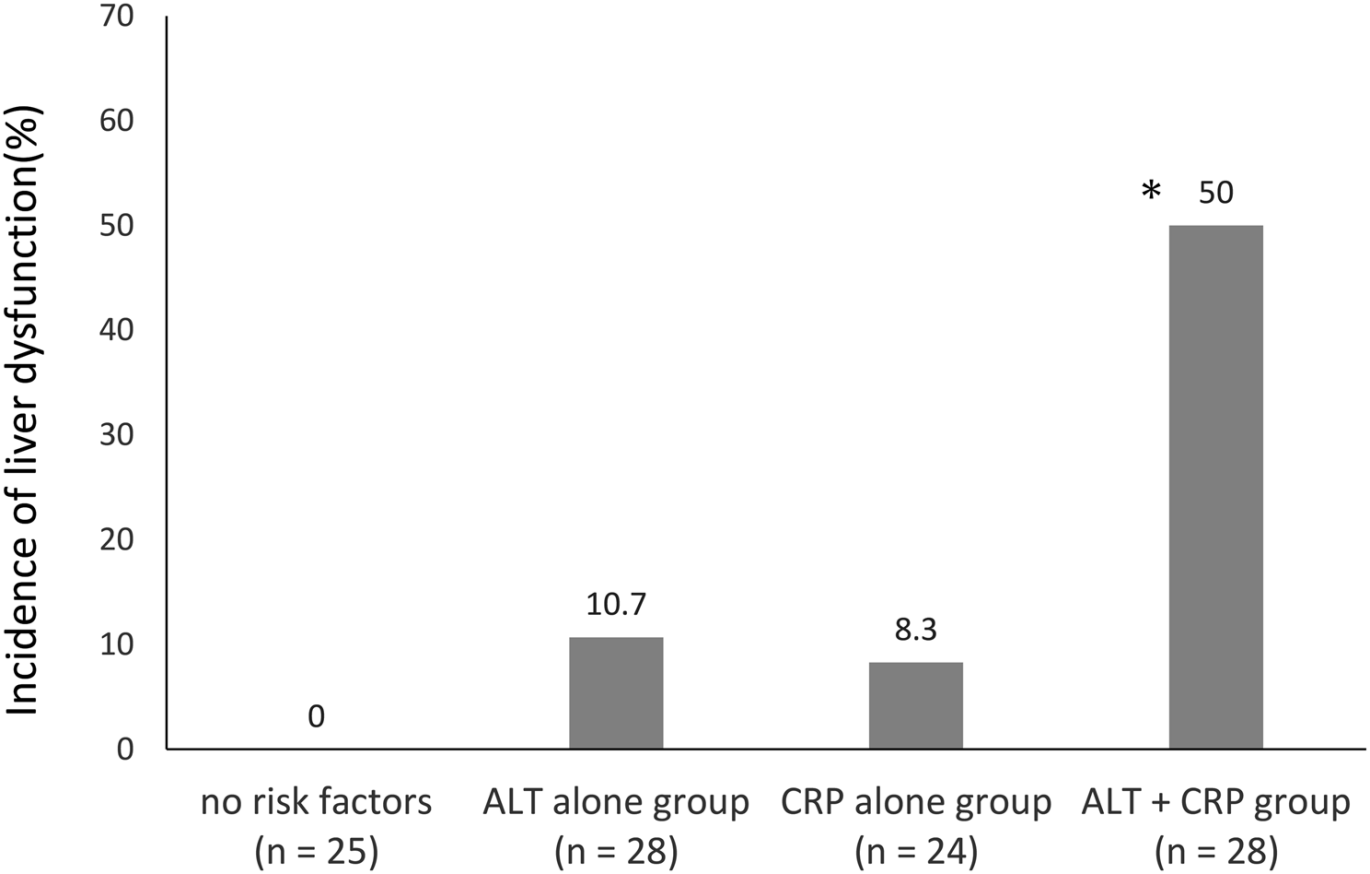
Incidence of liver dysfunction according to the number of risk factors. *p < 0.001 (Fisher’s exact test with Bonferroni correction vs. each of the other three groups)

ALT and CRP were dichotomized based on their respective cutoff values, and the incidence of liver dysfunction was calculated according to the number of risk factors present. The incidence rates were 0% (0/25) in patients with no risk factors, 10.7% (3/28) in those with elevated ALT only, 8.3% (2/24) in those with elevated CRP only, and 50% (14/28) in those with both elevated ALT and CRP. Patients with both risk factors exhibited a significantly higher incidence of liver dysfunction compared with each of the other groups, (all *p* < 0.001 Fig. 2).

## Discussion

No standardized definition of liver dysfunction exists across studies, and the assessment criteria vary depending on the study. In this study, patients who developed an ALT elevation of Grade 1 or higher according to CTCAE v5.0-JCOG after CTRX administration were defined as having liver dysfunction. In general, mild liver dysfunction corresponding to Grade 1 in CTCAE v5.0-JCOG does not necessarily require clinical intervention. However, even mild liver dysfunction may lead to discontinuation or modification of CTRX at the physician’s discretion, potentially affecting treatment; therefore, this definition was adopted. In fact, in our study, there were three cases wherein CTRX was discontinued or switched to another antibiotic due to a Grade 1 ALT elevation after CTRX administration, at the physician’s discretion. Only one patient developed a Grade 2 or higher ALT elevation, making stratified analysis according to severity difficult.

Nakaharai et al. identified a CTRX dose of 4 g/day as a risk factor for liver dysfunction [2]. In the present study, only three patients received a dose of 4 g/day, and liver dysfunction occurred in one of them. However, given the extremely small sample size, a meaningful statistical evaluation of dose-related risk was not feasible. therefore, the impact of dosage on liver dysfunction incidence in this study was considered limited. Regarding the duration of CTRX administration, previous studies have adopted different inclusion criteria, such as ≥ 2 days of treatment [3] or ≥ 5 days [2, 4]. However, Feng et al. reported that CTRX-associated liver dysfunction can occur as early as 0.5 days after treatment initiation [8]. Therefore, in this study, all patients who received CTRX were included in the analysis, regardless of the duration of administration.

No significant difference was observed in the cumulative dose of CTRX between the two groups; however, this may be because the majority of patients received 2 g/day and there was no significant difference in the duration of administration in this study. Feng et al. reported that the time to onset of liver dysfunction ranged from 0.5 to 47 days [8]. In our study, the median time to onset was 8 days, which falls within the previously reported range. This supports that the liver dysfunction observed in this study was attributable to CTRX administration.

Barman et al. reported a significant association between CTRX-associated liver dysfunction and concomitant use of hepatically metabolized drugs, such as acetaminophen and azithromycin [3]. However, no such association was observed in our study. The median age of our study was in the late 80s, and it is possible that the doses of acetaminophen administered in combination were lower than the typical adult doses, which may have contributed to this discrepancy. Furthermore, only one patient in our study received azithromycin, precluding meaningful analysis.

Regarding age, although this study included only older patients aged ≥ 65 years, the median age was in the late 80s and the age distribution was relatively uniform. Therefore, it was difficult to detect a risk gradient according to age. Consequently, the findings of this study strongly suggest the characteristics of CTRX-associated liver dysfunction in this age group.

To ensure a uniform baseline patients with ALT levels exceeding Grade 1 immediately prior to CTRX administration were excluded. Patients who were switched to CTRX from other antibiotics were also excluded to accurately assess baseline inflammation status prior to CTRX administration.

Univariate and multivariate logistic regression analyses identified pre-treatment ALT level as an independent risk factor for liver dysfunction, with a cutoff value of 11 U/L, which lies within the normal range. However, Dong et al. demonstrated that ALT levels decline with age compared with those in younger adults [9], suggesting that even relatively modest elevations within the normal range may be clinically meaningful in older individuals. Thus, ALT levels within the normal range may still represent a risk factor for CTRX-associated liver dysfunction in older patients. and the risk may be even greater when ALT levels exceed the normal range prior to CTRX administration.

CRP showed borderline statistical significance, with the 95% CI for the OR including 1.00. CRP production is initiated when pro-inflammatory cytokines such as tumor necrosis factor-α and interleukin-1β, generated locally during infection, stimulate Kupffer cells to produce interleukin-6 (IL-6), which in turn induces CRP synthesis in hepatocytes [10]. Because CRP levels correlate with the intensity of the inflammatory response, high CRP levels can be considered a surrogate marker of systemic inflammatory burden, reflecting elevated IL-6 levels as well. Kato et al. reported that drug transporters, including multidrug resistance-associated protein 2 (MRP-2) and breast cancer resistance protein (BCRP), contribute to the biliary excretion of β-lactam antibiotics, including CTRX [11]. Furthermore, Lee et al. demonstrated that IL-6 downregulates the expression of MRP-2 and BCRP in hepatocytes [12]. Collectively, these findings suggest that infection-induced IL-6 may impair the biliary excretion of CTRX by downregulating relevant drug transporter expression, thereby increasing intracellular drug accumulation and potentially precipitating liver dysfunction. When decreased biliary excretion due to elevated IL-6 coexists with relatively high baseline ALT levels, the risk of liver dysfunction may be further amplified. Using the identified cutoff values for ALT and CRP, both variables were dichotomized, and the incidence of liver dysfunction was compared according to the number of risk factors present. Patients with both elevated ALT and CRP levels exhibited an incidence of 50%, which was significantly higher than that observed in the other groups. These findings suggest that careful monitoring of liver function is warranted in patients with ALT ≥ 11 U/L and CRP ≥ 5.9 mg/dL prior to CTRX administration.

Previous studies in adult populations including younger patients [2–4] reported administration of high-doses (4 g/day), concomitant use of hepatically metabolized drugs, and an association with the albumin–bilirubin score as risk factors for CTRX-associated liver dysfunction; however, in older patients, who were the focus of this study, the effects of dosage, duration of administration, and concomitant medications were limited. Furthermore, the aim of this study was to explore risk factors for liver dysfunction using routinely available clinical data. Therefore, the albumin–bilirubin score was not included in the analysis. In contrast, this study demonstrated that routinely measured laboratory parameters, such as ALT and CRP, may serve as risk factors. These findings suggest that risk assessment indicators for CTRX-associated liver dysfunction may differ between younger/adult patients and older patients.

This study has several limitations. First, liver dysfunction was evaluated solely based on ALT levels, which may be insufficient as a definition of liver dysfunction. Second, this was a small, single-center, retrospective study with a limited sample size, which precluded sufficient analysis of factors other than ALT and CRP. Therefore, the present analysis should be regarded as exploratory and hypothesis-generating. Future studies with larger sample sizes are needed to further evaluate other potential confounders, including the severity of infection, concomitant medications other than acetaminophen, CTRX dosage, duration of administration, and cumulative dose.

CTRX is also known to cause biliary pseudolithiasis as a specific adverse effect. Although this was not analyzed in the present study, one patient was found to have gallstones on imaging after CTRX administration. However, biliary imaging is generally performed based on clinical symptoms, and most patients did not undergo imaging; therefore, the true incidence of biliary pseudolithiasis in this study remains unknown. On the other hand, several reports have described cases of biliary pseudolithiasis accompanied by elevations in AST and ALT [13–15], suggesting that undetected cases of biliary pseudolithiasis may have been present in our study population. Accordingly, the association between biliary pseudolithiasis and liver dysfunction in older patients warrants further investigation.

## Conclusion

The result of this study suggests that, among patients aged ≥ 65 years, those with both ALT ≥ 11 U/L and CRP ≥ 5.9 mg/dL prior to CTRX administration may be at increased risk of liver dysfunction. Accordingly, careful monitoring of liver function during and after CTRX administration is recommended.

## Data Availability

The datasets generated and analyzed during the current study are available from the corresponding author upon reasonable request.

## Abbreviations

ALT: Alanine aminotransferase
ALP: Alkaline phosphatase
AST: Aspartate aminotransferase
AUC: Area under the curve
BCRP: Breast cancer resistance protein
CI: Confidence interval
CRP: C-reactive protein
CTCAE v5.0-JCOG: Common Terminology Criteria for Adverse Events version 5.0 Japanese Clinical Oncology Group
CTRX: Ceftriaxone
MRP-2: Multidrug resistance–associated protein 2
OR: Odds ratio
ROC: Receiver operating characteristic
T-Bil: Total bilirubin
ULN: Upper limit of normal
VIF: Variance inflation factor
